# Association between cardiovascular risk and ADAM10 in cognitively healthy older adults

**DOI:** 10.1002/alz70856_104801

**Published:** 2026-01-07

**Authors:** Pedro Henrique Moreira Victoriano, Vanessa Alexandre da Silva, Marina Mantellatto Grigoli, Sabrina Dorta de Oliveira, Cecilia Patricia Popolin, Lucas Nogueira de Carvalho Pelegrini, Ari Alex Ramos, Márcia Regina Cominetti

**Affiliations:** ^1^ Federal University of São Carlos, São Carlos, SP, Brazil; ^2^ Federal University of São Carlos, São Carlos, São Paulo, Brazil; ^3^ Universidade Federal de São Paulo (UNIFESP), São Paulo, São Paulo, Brazil

## Abstract

**Background:**

Cardiovascular risk factors (CVRF) have been strongly implicated in elevating the risk of developing Alzheimer's disease (AD), highlighting the intricate relationship between cardiovascular health and neurodegeneration. In this context, ADAM10 emerges as a promising blood‐based biomarker for AD, given its well‐established association with amyloid deposition and its potential to provide insights into the link between cardiovascular health and amyloid pathology. This study explores the potential relationship between CVRF and plasma ADAM10 levels in cognitively healthy older adults.

**Method:**

In this cross‐sectional analysis, the cognitive function was assessed using the Addenbrooke's Cognitive Examination‐Revised (ACE‐R). The Framingham Score determined the cardiovascular risk profile. ADAM10 plasma levels were measured using Enzyme‐linked immunosorbent assays (ELISA). Group comparisons were conducted using the Wilcoxon signed‐rank test for continuous variables and the Chi‐square test for categorical variables. Linear regression models were employed to investigate associations with cognitive outcomes.

**Result:**

The study included 84 adults aged 60 and older, from whom 52 participants (96.2% females) were assigned to the low cardiovascular risk group, whereas 33 participants (15.2% females) were classified as medium/high cardiovascular risk. Despite the robust strength of the association between ADAM10 plasma levels and cognitive scores in non‐adjusted analyses (*β* = 0.162, 95% CI [0.008, 0.316], *P* = .040), this association did not remain statistically significant in the mutually adjusted model (*β* = 0.077, 95% CI [‐0.048, 0.202], *P* = .226). As expected, multivariate analyses revealed that older age (*β* = ‐0.465, 95% CI [‐0.767, ‐0.162], *P* = .003) was significantly associated with worse cognitive performance. Conversely, higher levels of formal education (*β* = 0.774, 95% CI [0.510, 1.038], *P* < .001) were associated with better cognition.

**Conclusion:**

No evidence of a robust association between ADAM10 levels and CVRF was found. The lack of a significant association may be attributed to the insufficient number of participants classified as having high CVR. Larger samples are needed to enable more detailed analyses with optimal group stratification. We speculate that the inclusion of participants with higher levels of formal education may have contributed to this non‐significant result, given the association between higher education and preventive health care.

